# Identification of New Allergens in Macadamia Nut and Cross-Reactivity with Other Tree Nuts in a Spanish Cohort

**DOI:** 10.3390/nu16070947

**Published:** 2024-03-26

**Authors:** Gloria Gutiérrez-Díaz, Diana Betancor, Jorge Parrón-Ballesteros, Rubén G. Gordo, Estela S. Castromil-Benito, Elisa Haroun, María Vázquez de la Torre, Javier Turnay, Mayte Villalba, Javier Cuesta-Herranz, Carlos Pastor-Vargas

**Affiliations:** 1 Department of Biochemistry and Molecular Biology, Universidad Complutense de Madrid, 28040 Madrid, Spain; glogut01@ucm.es (G.G.-D.); jparron@ucm.es (J.P.-B.); rugarc09@ucm.es (R.G.G.); estelasc@ucm.es (E.S.C.-B.); turnay@ucm.es (J.T.); mvillalb@ucm.es (M.V.); 2 Department of Allergy and Immunology, IIS-Fundación Jiménez Díaz, Universidad Autónoma de Madrid (UAM), 28040 Madrid, Spain; diana13_b@hotmail.com (D.B.); j.cuestaherranz@gmail.com (J.C.-H.); 3 Department of Allergy, Hospital Universitario Infanta Leonor, 28040 Madrid, Spain; elisaharoun@hotmail.com (E.H.); mvazquezt@salud.madrid.org (M.V.d.l.T.); 4 Redes de Investigación Cooperativa Orientadas a Resultados en Salud (RICORS) Red de Enfermedades Inflamatorias (REI)—RD21/0002/0028, Instituto de Salud Carlos III, 28029 Madrid, Spain; 5 Red de Asma, Reacciones Adversas y Alérgicas (ARADyAL)—RD16/0006/0013, Instituto de Salud Carlos III, 28029 Madrid, Spain

**Keywords:** macadamia nut, tree nut allergy, oleosin, aspartyl protease, pectin acetylesterase

## Abstract

The consumption of macadamia nuts has increased due to their cardioprotective and antioxidant properties. However, this rise is consistent with an increase in the cases of macadamia nut allergy, leading to severe reactions. Although two *Macadamia integrifolia* allergens (Mac i 1 and Mac i 2) have been identified in Australian and Japanese patients, the allergenic sensitization patterns in Western European populations, particularly in Spain, remain unclear. For this purpose, seven patients with macadamia nut allergy were recruited in Spain. Macadamia nut protein extracts were prepared and, together with hazelnut and walnut extracts, were used in Western blot and inhibition assays. IgE-reactive proteins were identified using MALDI-TOF/TOF mass spectrometry (MS). Immunoblotting assays revealed various IgE-binding proteins in macadamia nut extracts. Mass spectrometry identified three new allergens: an oleosin, a pectin acetylesterase, and an aspartyl protease. Cross-reactivity studies showed that hazelnut extract but not walnut extract inhibited macadamia nut oleosin-specific IgE binding. This suggests that oleosin could be used as marker for macadamia–hazelnut cross-reactivity. The results show an allergenic profile in the Spanish cohort different from that previously detected in Australian and Japanese populations. The distinct sensitization profiles observed highlight the potential influence of dietary habits and environmental factors exposure on allergenicity.

## 1. Introduction

Macadamia nuts are edible seeds from *Macadamia tetraphylla* and *Macadamia integrifolia* trees, both native to Australia, the latter being the commercial variety available worldwide [1]. Macadamia nuts are known to have benefits for human health, in particular for their cardioprotective [2] and antioxidant [3] properties, due to their abundance of unsaturated fatty acids, polyphenols, and carotenoids. Therefore, they have been introduced into the diets of Western countries in recent years. Along with the increase in their consumption, there has been an increase in reported cases of macadamia nut allergies, with most patients suffering from severe allergic reactions [4,5]. However, macadamia allergy is an emerging pathology, and to date, there are no data for its prevalence in wide populations.

The clinical relevance of these allergies highlights the need for further research into their allergenic components. To date, only two allergens have been described in *Macadamia integrifolia*: Mac i 1 (vicilin) and Mac i 2 (11S globulin) as well as antimicrobial peptides derived from Mac i 1 processing [6,7]. Tree nut allergy is a condition suffered by a range of 1–3% of the general population, reaching prevalence up to 4.9% in determinate geographical areas [8]. IgE cross-reactivity between macadamia nut and other nut extracts has been reported [9]. Nevertheless, the precise allergens involved in these cross-reactions remain to be elucidated.

All the studies mentioned above were performed in Japanese and Australian patients. However, geographical differences (including factors such as climate, humidity, or the pollinosis present in the region) are known to impact the prevalence of allergies to different foods and even the molecular sensitization pattern in patients allergic to the same foods [10,11].

Understanding the prevalence and molecular pattern of macadamia nut allergy may also contribute to public health initiatives [12]. By identifying the specific allergens responsible for adverse reactions, treatment strategies, including immunotherapy, can be better customized. Moreover, awareness of macadamia nut allergy and its possible cross-reactivity with other nuts may enable individuals to reduce the risk of adverse reactions [13].

For this reason, the aim of this study was to evaluate the molecular profile of allergen sensitization in different populations, particularly in a cohort of Spanish macadamia nut allergic patients, along with the identification of the involved allergens and possible cross-reactivity with other tree nuts. 

## 2. Materials and Methods

### 2.1. Preparation and Quantification of Macadamia Nut Protein Extract

Macadamia extract was prepared as previously reported [6]. The extract was prepared using raw macadamia nut because the patients recruited experienced symptoms after raw nut ingestion. Briefly, raw macadamia nuts (20 g from species *M. integrifolia*, the commercially available variety in Spain) from a local supermarket were ground in the presence of liquid nitrogen, homogenized in 100 mL of phosphate-buffered saline (PBS) (1:5, *w*/*v*), and stirred overnight at 4 °C. The extract was centrifuged three times for 30 min at 4 °C and 10,000 rpm and dialyzed against 0.1 M ammonium bicarbonate at 4 °C under agitation, using a 3.5 kDa dialysis membrane (Spectra-Por, Gardena, CA, USA). Quantification was performed by the Bradford method using Coomassie Plus reagent (Pierce, Rockford, IL, USA). Extracts were lyophilized, resuspended in PBS, and centrifuged for 14 min at 4 °C and 13,000 rpm. Finally, the supernatants were collected and stored at −20 °C. 

### 2.2. Electrophoretic Procedures for Protein Characterization

Macadamia nut extract (25 μg/lane) was separated by SDS-PAGE under reducing and non-reducing conditions, using Mini-Protean II systems (Bio-Rad, Hercules, CA, USA) and 15% polyacrylamide gels. The separated proteins were then either stained with Coomassie Brilliant Blue R-250 (Merk, Darmstadt, Germany) or transferred to a nitrocellulose membrane. Precision Plus Protein^TM^ Dual Color (Bio-Rad) molecular mass leaders were used as reference. 

### 2.3. Sera Samples from Allergic Patients

Macadamia nut allergy was diagnosed according to the criteria reported by food allergy committee from SEAIC (www.seaic.org, accessed on 29 January 2024). All the patients reported a suggestive clinical history of immediate allergic reaction after consuming macadamia nuts, positive skin prick-by-prick tests, and/or specific IgE levels for raw macadamia nut. The recorded data included demographic characteristics, symptoms of the allergic reaction, presence of previous atopy based on positive skin tests to common aeroallergens, and allergy to other nuts. Total and specific serum IgE to macadamia nut, walnut (*Juglans regia*) and hazelnut (*Corylus avellana*) proteins were determined using ImmunoCAP (ThermoFisher Scientific, Rockford, IL, USA), with values above 0.35 kUA/L considered positive. Prick-by-prick was considered positive if the diameter of the wheal was at least 3 mm larger than that of the negative controls. A serum sample was collected from each patient and stored at −20 °C until use.

For the statistical analysis, qualitative variables were expressed as percentages, and confidence intervals were calculated at 95%. For quantitative variables, means and standard deviation (SD) were calculated, and for specific IgE results, medians and 25th (Q1) and 75th (Q3) percentiles were given. Statistical analysis was performed using GraphPadInstat 6.0 version (GraphPadSoftware Inc., San Diego, CA, USA). The Ethics Committee of both hospitals approved the study, and all patients provided written informed consent.

### 2.4. Immunological Assays with Patients’ Sera

Extracts (25 μg of extract/lane) were blotted onto nitrocellulose membranes (Amersham) after SDS-PAGE. Firstly, membrane strips were blocked for 2 h with BSA and low-fat powdered milk (both 3% (*w*/*v*)) diluted in PBS containing 0.1% (*v*/*v*) Tween-20 (PBS-T). Subsequently, the strips were incubated overnight at 4 °C under agitation, with individual sera of macadamia nut allergic patients or non-atopic serum as negative control (diluted 1/3 and 1/10, respectively, in blocking buffer). In the case of the Western blot for protein identification by mass spectrometry, a pool of sera diluted 1/3 in blocking buffer was used. For detection of binding human IgE, the strips were incubated for 1 h at room temperature with a horseradish peroxidase-labelled mouse anti-human IgE monoclonal antibody (Southern Biotech, Birmingham, AL, USA) diluted 1/2000 in PBS-T 0.05% (*v*/*v*) with low-fat powdered milk (1.5% (*w*/*v*)). The chemiluminescent signal was developed by Clarity Western ECL reagent (Bio-Rad) and detected in a luminescent image analyzer LAS3000 (Fujifilm, Tokio, Japan), with an exposure time of 30 min.

Inhibition assays were performed to identify IgE-mediated cross-reactivity. Sera were pre-incubated with 100 µg inhibitor/mL for 4 h at room temperature with stirring. At the end, the samples were centrifuged for 10 min at 4 °C and 13,000 rpm, and inhibited sera were used for Western blot.

### 2.5. Protein Identification by LC-MS/MS

IgE-reactive proteins were identified using MALDI-TOF/TOF mass spectrometry (MS) based on liquid chromatography MS in tandem (LC-MS/MS), at the Proteomics Unit of the Universidad Complutense of Madrid. To avoid contamination, protein bands were cut with a sterile scalpel and preserved in ultrapure H_2_O milli-Q at 4 °C. Trypsin was used to digest each protein, generating peptides of a size more suitable for subsequent mass spectrometry. The set of peptides of different sizes generated constitutes the peptide *fingerprint*, which is unique and exclusive for each protein. The analysis was performed in Bruker ULTRAFLEX MALDI-TOF/TOF (Bruker-Daltonics, Bremen, Germanyequipment, with which the peptide *fingerprint*, molecular mass, and isoelectric points (pI) were obtained by searching on non-redundant protein sequence databases, concluding with the proteins in question. 

## 3. Results 

### 3.1. Patient Clinical Characterization

Seven patients allergic to macadamia nut were recruited at Fundación Jiménez Díaz Hospital or Infanta Leonor University Hospital, Madrid (Spain). The mean age of the patients was 33.4 years (range 9–58 years); four of them (57.1%) were female. Prick-by-prick test with raw macadamia was positive in all patients. The median specific IgE concentration for macadamia nut was 0.81 kUA/L (Q1–Q3 percentiles: 0.11–5.71 kU/L). Detailed information is given in Table 1.

### 3.2. Immunodetection of IgE-Reactive Proteins in Macadamia Nut, Hazelnut, and Walnut Extracts by Western Blot

**Figure 1:**
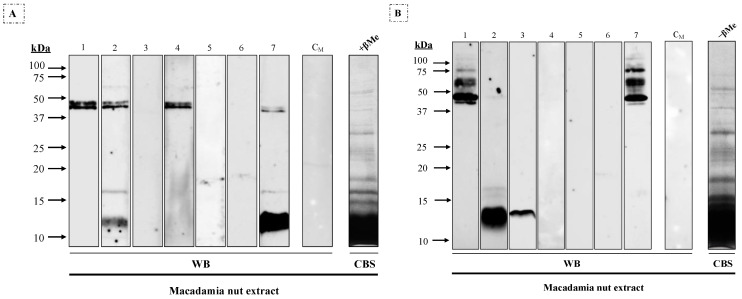
Western blot using 1/3 diluted sera from seven patients allergic to macadamia nut against macadamia nut extract (25 μg/lane) under (**A**) reducing (+βMe) and (**B**) non-reducing (−βMe) conditions. C_M_, serum from a non-atopic control patient; CBS, Coomassie Blue staining of the macadamia nut extract.

Different molecular mass IgE-binding bands were observed in macadamia extract under reducing conditions (Figure 1A) after immunoblotting assays with individual sera: 12–16 kDa (#2 and #7) and a double band around 45 kDa (#1, #2, #4 and #7). In non-reducing conditions (Figure 1B), multiple bands between 40 to 75 kDa were observed in two patients (#1 and #7), along with 14 kDa (#2 and #3) and 16 kDa (#2).

**Figure 2:**
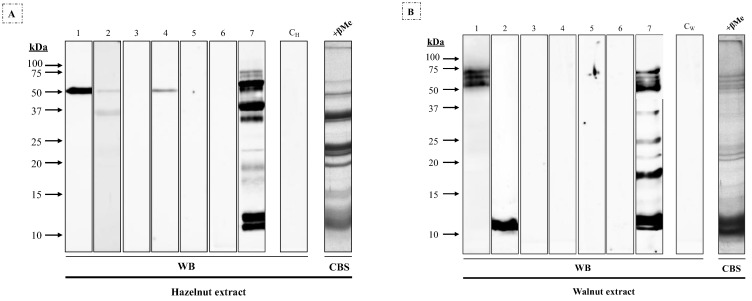
Western blot using 1/3 diluted sera from seven patients allergic to macadamia nut against (**A**) hazelnut and (**B**) walnut extracts (25 μg/row) under reducing conditions. C_H_ and C_W_, sera from non-atopic control patients; CBS, Coomassie Blue staining of (**A**) hazelnut and (**B**) walnut extracts.

This pattern was related to that observed when these sera were incubated with reduced hazelnut extract (Figure 2A), where patients #1, #2, #4, and #7 showed a 45 kDa band; and walnut extract (Figure 2B), where patients #1 and #7 yielded a high-molecular-mass triplet and #2 and #7 a 12 kDa band, along with non-coincident bands between 10 and 50 kDa.

### 3.3. Evaluation of Cross-Reactivity between Macadamia Nut and Hazelnut and Walnut Extracts by Western Blot

**Figure 3:**
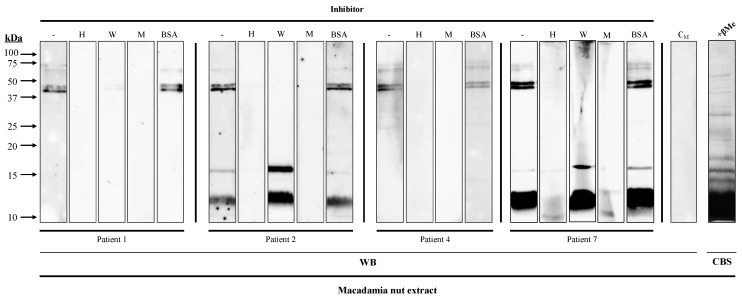
Inhibition Western blot of IgE recognition from four macadamia-nut-allergic patients’ 1/3 diluted sera, using hazelnut (H) and walnut (W) extracts as inhibitors, macadamia nut extract (M) as positive control, and BSA as negative control. C_M_, serum from a non-atopic control patient; CBS, Coomassie Blue staining of macadamia nut extracts.

IgE binding to the 45 kDa band from macadamia extract was inhibited with hazelnut and walnut extracts in all the patients, whereas the 12 kDa and 16 kDa bands were inhibited exclusively with hazelnut extract in patients #2 and #7 (Figure 3).

### 3.4. Identification of IgE-Binding Proteins

**Figure 4:**
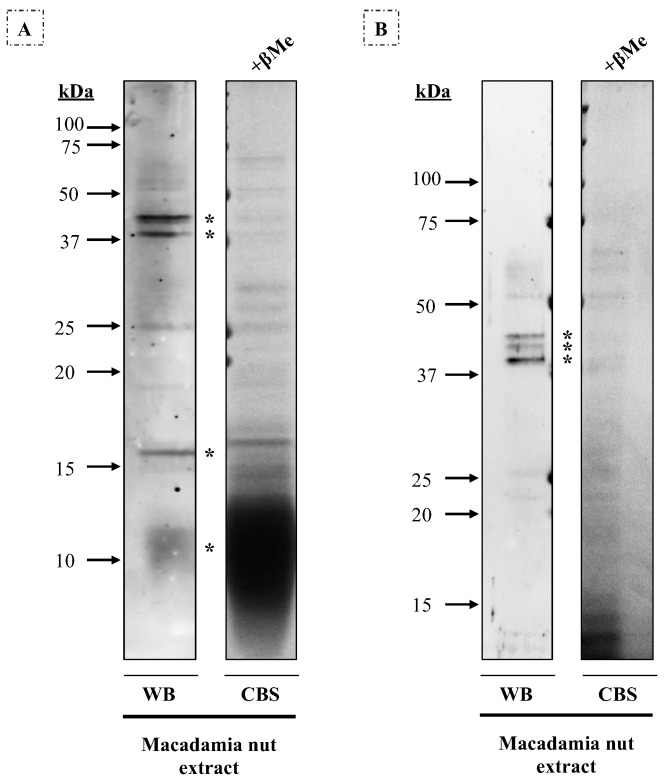
Western blot with macadamia nut extract (80 μg/row) under reducing conditions, using a 1/3 diluted pool of sera from seven patients allergic to macadamia nut. CBS, Coomassie Blue staining of the macadamia nut extract in (**A**) 14% and (**B**) 10% acrylamide gels. Protein bands of 12 kDa, 16 kDa, 40 kDa, and 45 kDa were extracted and identified on gel A, while on gel B, bands of about 40–42–45 kDa were detected. These protein bands are indicated with an asterisk.

Bands were extracted from the gels (Figure 4), and three allergenic proteins were identified by mass spectrometry. On gel A (14% acrylamide), the 12 kDa band corresponded to antimicrobial peptide 1, the 16 kDa band to oleosin, the 40 kDa band to a pectin acetylesterase X2 isoform, and the 45 kDa band to two different proteins: Mac i 1 (vicilin) and aspartyl protease AED3. On gel B (10% acrylamide), these results were verified: the 40 kDa band corresponded to pectin acetylesterase X2 isoform and the 42–45 kDa bands to aspartyl protease AED3.

Therefore, patients #1, #2, #4, and #7 (57.1%) are sensitized either to vicilin or aspartyl protease and pectin acetylesterase, whereas patients #2 and #7 (28.6%) are sensitized to oleosin and antimicrobial peptide 1. Although it cannot be ruled out that the allergenicity of these patients could be attributed to other bands not determined in the MS analysis, it is interesting that patients #2 and #7 are the only ones that reported allergy to walnut and hazelnut.

## 4. Discussion

Macadamia nut allergy is an increasingly common condition. Currently, only 11S globulin, vicilin, and antimicrobial peptide 1 have been identified as allergens for Australian and Japanese patients. In this report, three new allergens (oleosin, pectin acetylesterase, and aspartyl protease) were identified in a Spanish cohort of patients using the same techniques that were used by Kabasser et al. [6]. Even though Mac i 1 and antimicrobial peptide sensitization were present in both Australian and Spanish populations, Mac i 2 sensitization did not appear in Spanish patients; in contrast, novel allergenic families were found. Although the study cohort is still small, and cohorts of larger patient would be needed to confirm these regional differences, these might be derived from different dietary habits and pollen exposure.

Oleosins are highly resistant, non-glycosylated proteins, whose relevance as an allergens family has been rising recently [14], as they have been identified in walnut [15], hazelnut (Cor a 12), peanut (Ara h 9), sesame seeds (Ses i 4), and Tartarian buckwheat (Fag t 6). Our results show that macadamia nut oleosin is cross-reactive with hazelnut but not walnut; however, oleosins are usually overlooked as allergens due to their affinity to lipid droplets. Therefore, low inhibition of walnut extract can be due to the absence of walnut oleosins in our extracts. These data suggest that oleosins are a cross-reactive allergen in macadamia nut.

Pectin esterases, pectin methylesterases, and aspartyl proteases play a role in cell wall remodeling, defense against pathogens, and abiotic stress in plants. Even though pectin acetylesterases had never been identified as allergens before, homologous pectin methylesterases are relevant allergens in kiwifruit (Act d 7) and pollens (Ole e 11 and Sal k 1), and their expression is increased in heat-stress conditions [16].

Aspartyl proteases are major allergens in lettuce (Lac s AP) [17] and have also been described as allergens in almond (Pru du AP), fungi, and insects. Even though the allergenic profile of macadamia nuts is not related to the characteristic profile of other tree nuts, the botanical differences between macadamia nuts (family *Proteaceae*) and the other described allergenic tree nuts must be considered. Therefore, protein families that are considered pan allergens (e.g., profilins) in tree nuts can be absent in *Macadamia integrifolia*, and protein families that are not described as allergenic in tree nuts can rise as novel allergens in this species.

In this regard, further research should be performed to identify the whole allergen profile of the macadamia nut and the role of macadamia sensitization in the context of tree nut allergy.

## 5. Conclusions

In conclusion, three new allergens in macadamia nut were identified in the largest series of macadamia-nut-allergic patients reported to date. Additionally, we confirmed the previously known sensitization to Mac i 1 and macadamia antimicrobial peptide 1. This study has revealed novel allergen families in tree nuts that are recognized after sensitization to macadamia nuts (aspartyl protease and pectin acetylesterase). These allergens may become more prevalent as macadamia nuts are introduced to the European population, along with the identification of oleosin as a predictive marker of macadamia–hazelnut cross-reactivity. 

## Figures and Tables

**Table 1:** Clinical features of macadamia nut allergic patients.

Number of Patient	Age	Sex	Atopy	Prick-by-Prick Macadamia Nut	IgE to Macadamia (kU/L)	Allergy Reaction	IgE to Walnut (kU/L)	IgE to Hazelnut (kU/L)	Allergy to Other Nuts
1	31	F	Yes	5 × 4	0.11	OAS	0	0	No
2	9	F	No	9 × 3	3.37	OAS + rhynoconjunctivitis	0.58	0.08	W, H
3	47	M	Yes	7 × 6	0.85	Syst.	0	0	No
4	35	F	Yes	8 × 7	0.49	Syst.	0	0	No
5	58	M	No	7 × 2	0.07	Syst.	0	0	No
6	41	M	No	6 × 4	0.81	Syst.	NA	0	No
7	13	F	Yes	10 × 8	5.71	OAS	70.5	24.4	W, H, P, C

F, female; M, male; OAS, oral allergy syndrome; Syst., systemic reaction; NA, not available; W, walnut; H, hazelnut; P, pistachio; C, cashew.

## Data Availability

The original contributions presented in the study are included in the article, further inquiries can be directed to the corresponding author.
